# Implementing the advantages of contrast-enhanced mammography and contrast-enhanced breast MRI in breast cancer staging

**DOI:** 10.1007/s00330-024-10896-0

**Published:** 2024-07-12

**Authors:** Eva M. Fallenberg

**Affiliations:** Department of Diagnostic and Interventional Radiology, TUM School of Medicine & Health, Klinikum Rechts der Isar, Technical University of Munich (TUM), Ismaninger Str. 22, 81675 München, Germany

Breast cancer is the most common cancer among women globally. It remains a significant health concern worldwide, affecting millions of individuals each year. The incidence varies across populations, emphasizing the importance of ongoing research and awareness campaigns to encourage early detection and accurate staging, as they are crucial for effective treatment and improved outcomes.

During the last decade, a significant increase and improvement in breast cancer detection and treatment have been achieved. In addition to imaging improvements, many new systemic drugs have been developed, enabling a more personalized treatment, for example, by addressing dedicated receptors or compromising vessel growth depending on the different tumor types and receptor status of breast cancer. Besides this, surgeries are becoming less invasive with more complex oncoplastic procedures to enable breast conservation even in patients with multiple lesions, optimizing the cosmetic outcome.

Correct detection and extent evaluation, as well as locoregional staging of the tumor extent and of axillary lymph nodes, are critical components in determining the extent of breast cancer and guiding treatment decisions. The TNM system, which considers tumor size, lymph node involvement, and metastasis, helps oncologists classify the disease stage. Staging provides valuable information that aids in tailoring a personalized treatment plan for each patient [1].

Proper pre-treatment evaluation also enables reaching the quality indicators of the Breast Cancer Care Guidelines, like a high rate of single operations and high rates of MRI staging prior to primary systemic treatment (PST), provided by the EUSOMA Working Group [2].

The sensitivity of conventional digital mammography alone is limited. Therefore, it must be supplemented by ultrasound, tomosynthesis, contrast-enhanced mammography (CEM), or contrast-enhanced breast MRI (CE-MRI). Especially the first two can be compromised by dense and mastopathic breast tissue. In this case, the contrast-enhanced imaging methods are more beneficial [3].

MRI has been the gold standard for contrast-enhanced breast imaging since the late 1980s. It provides detailed images of breast tissue. Its high sensitivity in detecting small lesions and its ability to assess lymph node involvement make it a valuable tool in breast cancer diagnosis and staging [4]. Integrating MRI into the diagnostic process contributes to a more comprehensive evaluation of the disease [5, 6].

Approximately 20 years later, CEM was introduced, emerging as a technique for enhancing the visibility of tumors and improving the estimation of the extent of disease [7]. By using contrast agents, both modalities improve the detection of lesions and help distinguish between benign and malignant findings. This enhances diagnostic accuracy and contributes to better-informed treatment decisions.

Monitoring the response to breast cancer treatment is crucial for adjusting therapeutic strategies and ensuring optimal outcomes. Contrast-enhanced imaging techniques such as CE-MRI and CEM play a vital role in assessing treatment efficacy by visualizing changes in tumor size, morphology, and vascularity [6]. This aids oncologists in making informed decisions regarding ongoing treatments, potentially preventing unnecessary interventions, or adjusting therapies for better results in case of progressive disease.

Proper extent evaluation is necessary not only in invasive cancers but also in ductal carcinoma in situ (DCIS), as it can be larger than initially expected due to uncalcified parts of the DCIS. As the non-calcified parts of DCIS are usually not visible in non-enhanced imaging methods or ultrasound, contrast enhancement can be the only hint indicating a larger extent. This change in size can lead to a significant change in operative management making it necessary to switch from breast-conserving surgery to mastectomy [8]. This important information must be communicated to the surgeon.

When comparing CEM and CE-MRI, the latter is superior, especially regarding small additional lesions [7]. The depiction of the contrast enhancement, in general, is lower in CEM due to the 2D nature of the technique whereas in MRI the effect of superimposing issue and summation effect is lower. In addition, CEM has limitations due to the limited field of view, especially close to the chest wall, superpositions, and non-enhancement. Nevertheless, the presence and appearance of anatomical findings in the low energy image are well known from our experience with mammography and must always be considered when interpreting CEM, not only the enhancement maps, to avoid missing non- or very little enhancing cancers (e.g. invasive lobular or mucinous carcinoma) or DCIS calcifications. Regarding calcifications, the use of CEM is beneficial, as the MRI is not able to properly visualize calcifications as the only sign of DCIS. Furthermore, axillary lymph nodes are not properly assessable with CEM, resulting in the need for supplemental ultrasound. CEM outperforms CE-MRI with regard to false positive rates [9]. In general, performance data are more heterogenous for CEM than for CE-MRI [10]. We expect to gain a more homogeneous image with incoming additional studies.

One of the biggest advantages of CEM is the potential availability compared to CE-MRI, especially in countries where the number of MRI scanners is limited, or the procedure is not reimbursable. In addition, it can be used if there are patient contraindications such as metallic implants without MRI-safety approval or patients who suffer from claustrophobia, both common issues in MRI.

In summary, the ongoing advancements in breast cancer diagnosis and staging, particularly through CEM and CE-MRI, have significantly improved our ability to detect and characterize tumors. The integration of these imaging techniques into routine clinical practice enhances the precision of diagnosis, facilitates accurate staging, and allows for effective treatment monitoring. Nevertheless, for optimal breast imaging, a wise combination of the most appropriate imaging methods for each patient and careful medical history evaluation are necessary to get the full picture. As research continues to push the boundaries of medical imaging, the outlook for breast cancer patients continues to improve, offering hope for better outcomes and quality of life.
